# Supplementary figures and images for: Genome-Wide Identification and Expression Analysis of TPS Gene Family in Liriodendron chinense
Source: Genes (Basel). 2023 Mar 22;14(3):770. doi: 10.3390/genes14030770 (PMC10048281; doi:10.3390/genes14030770)

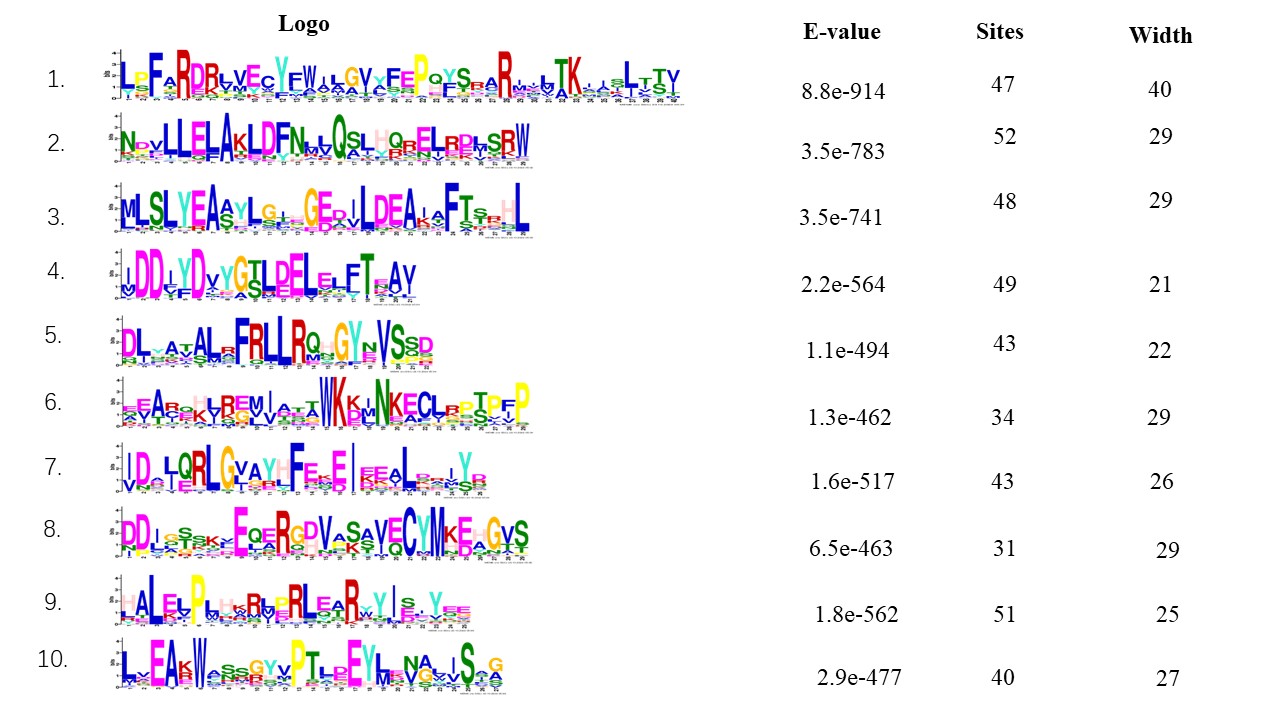

Supplement: Supplementary file 1 [file genes-14-00770-s001.zip › Figure S1.jpg]

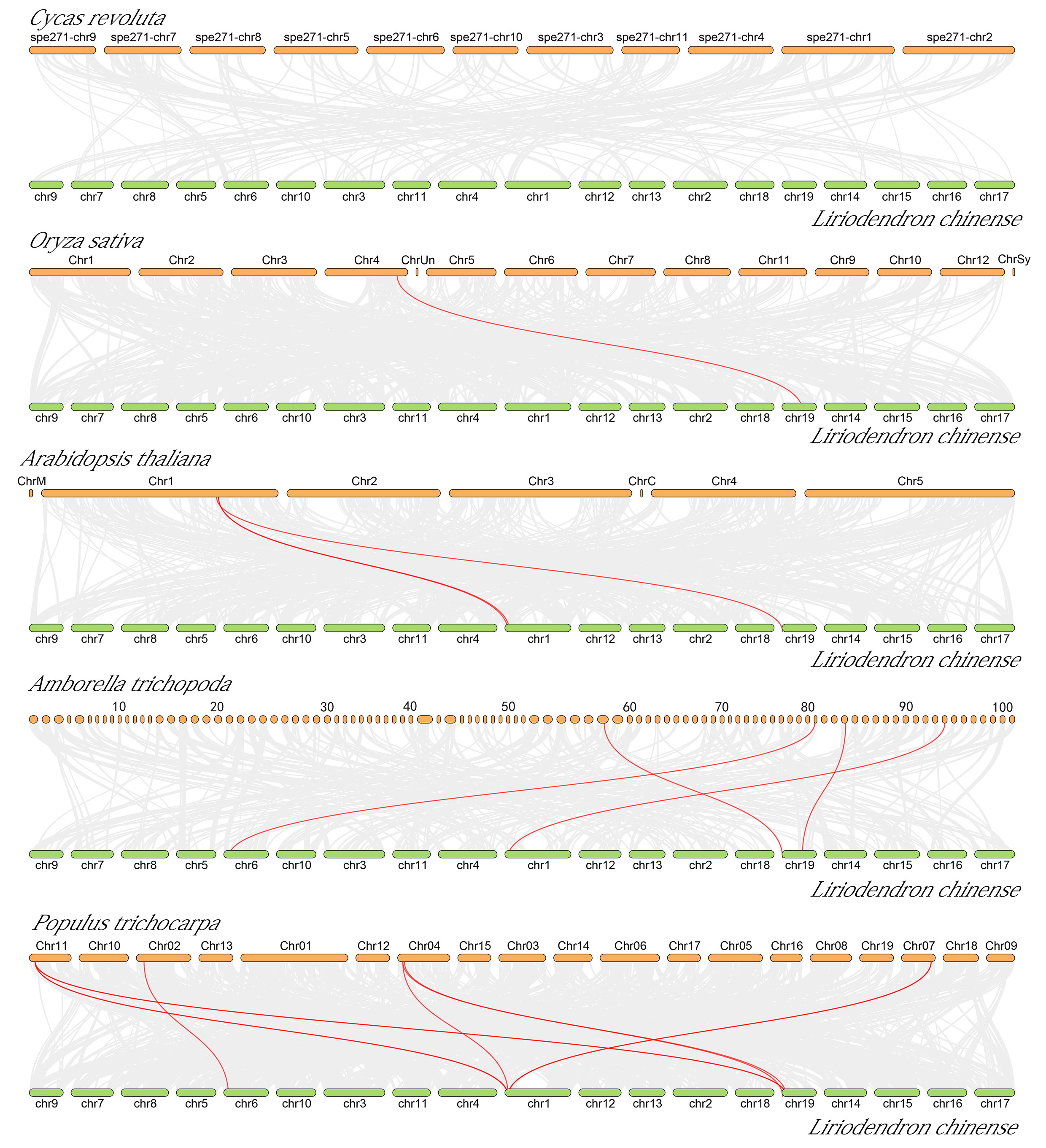

Supplement: Supplementary file 1 [file genes-14-00770-s001.zip › Figure S2.jpg]

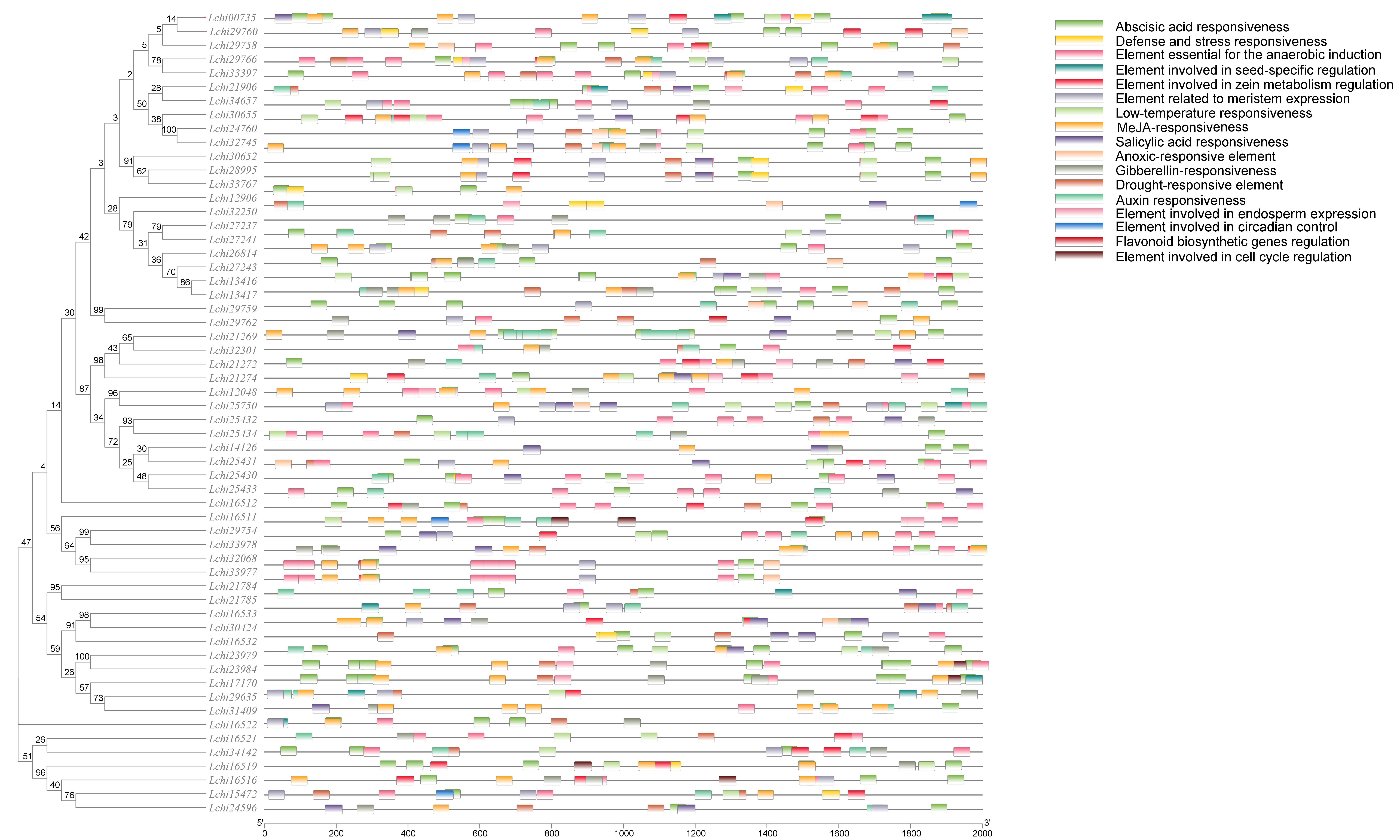

Supplement: Supplementary file 1 [file genes-14-00770-s001.zip › Figure S3.jpg]

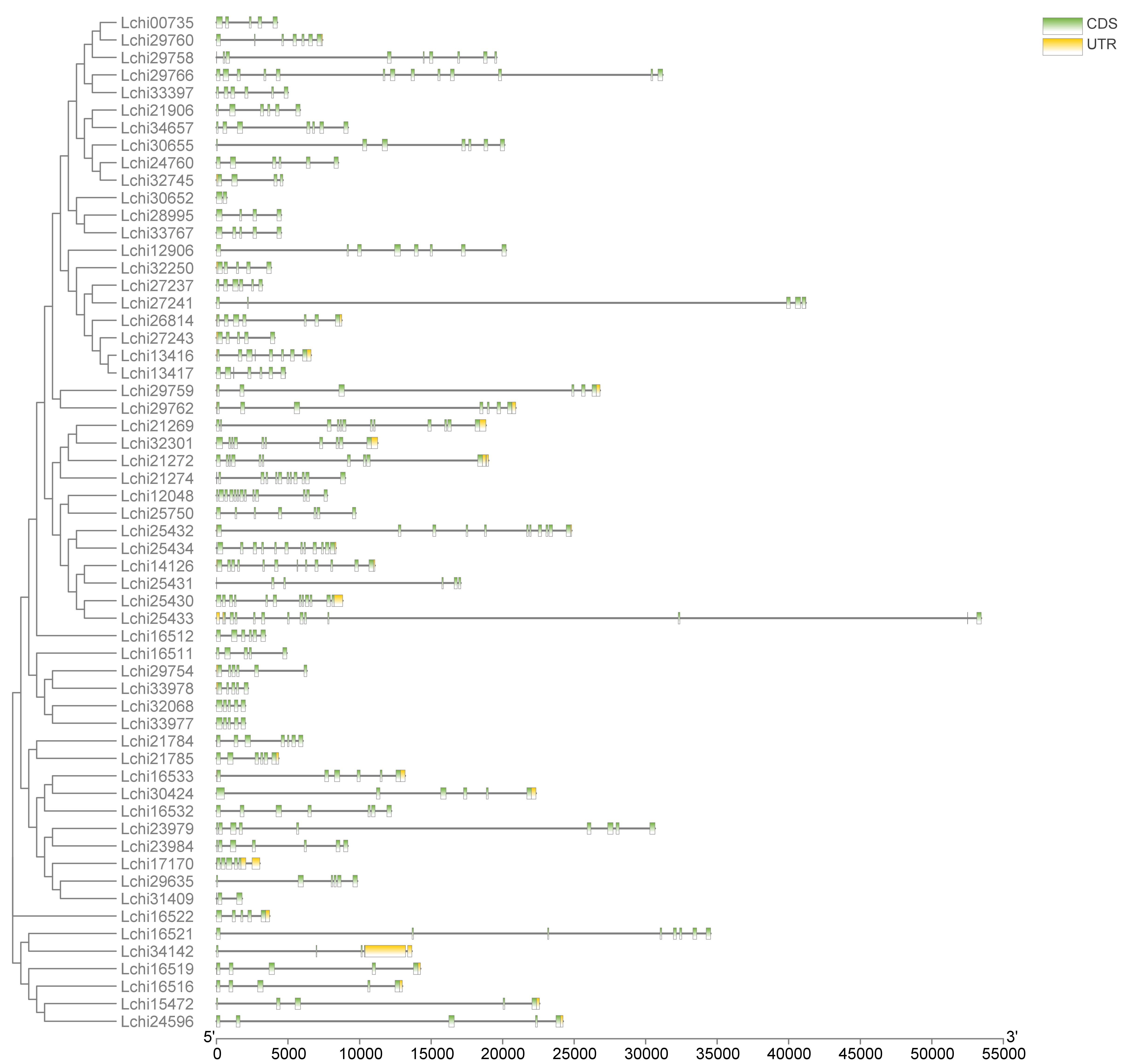

Supplement: Supplementary file 1 [file genes-14-00770-s001.zip › Figure S4.jpg]
